# Development of a Multiplex Quantitative Polymerase Chain Reaction Assay for the Detection of Duck Enteritis Virus, Goose Parvovirus, and Muscovy Duck Parvovirus

**DOI:** 10.3390/ani15111599

**Published:** 2025-05-29

**Authors:** Qian Qiu, Ruiming Hu, Zirui Liu, Linjie Yan, Fan Yang, Xueyan Dai, Chenghong Xing, Huabin Cao

**Affiliations:** 1 Jiangxi Provincial Key Laboratory for Animal Health, Institute of Animal Population Health, College of Animal Science and Technology, Jiangxi Agricultural University, No. 1101 Zhimin Avenue, Economic and Technological Development District, Nanchang 330045, China; 2 Department of Preventive Veterinary Medicine, College of Veterinary Medicine, Northwest A&F University, No. 22 Xinong Road, Yangling District, Xianyang 712000, China

**Keywords:** duck enteritis virus, goose parvovirus, muscovy duck parvovirus, multi-quantitative qPCR, TaqMan probe

## Abstract

Duck enteritis virus (DEV), goose parvovirus (GPV), and muscovy duck parvovirus (MDPV) all have similar symptoms after infection, such as severe diarrhea, which seriously affects the healthy development of the waterfowl industry. Hence, it is important to devise a rapid and precise assay for the detection of these three viruses. In this study, a TaqMan probe-based multi-quantitative polymerase chain reaction (qPCR) assay was developed and optimized. Three specific primers and probes were designed according to the conserved regions of UL6 of DEV, REP of GPV, and VP1 of MDPV, respectively. DEV demonstrated a detection limit of 11.6 copies, GPV detected a limit of 95 copies, and MDPV showcased a detection limit of 14.8 copies. The correlation coefficient is greater than 0.99, and the amplification efficiency is 89% to 93%. These results indicate that the multiplex qPCR assay has high sensitivity, specificity, and stability. Of the 215 clinical samples used in this study, 33 tested DEV positive, 25 tested GPV positive, and 24 tested MDPV positive. Overall, the assay established in the current study presents a rapid, efficient, specific, and sensitive tool for of detecting DEV, GPV, and MDPV.

## Simple Summary

Duck enteritis virus (DEV), goose parvovirus (GPV), and muscovy duck parvovirus (MDPV) cause severe diarrhea in waterfowl, leading to significant economic losses in the poultry industry. Current diagnostic challenges arise from similar symptoms, necessitating a rapid, precise method to distinguish these pathogens. This study developed a TaqMan probe-based multiplex qPCR assay targeting conserved regions of DEV, GPV, and MDPV. The assay demonstrated high sensitivity detection limits: 11.6 copies for DEV; 95 copies for GPV; 14.8 copies for MDPV; specificity and stability, with correlation coefficients above 0.99; and amplification efficiencies of 89–93%. Clinical testing of 215 samples identified the following: 33 tested DEV positive, 25 tested GPV positive, and 24 tested MDPV positive. This reliable, efficient tool enables simultaneous detection of all three viruses, aiding timely disease management.

## 1. Introduction

Since 2012, despite vaccination, cases of duck enteritis virus (DEV) infection have persisted in Shandong Province, China, causing economic losses to the duck farming industry [1]. DEV, a herpesvirus, is a double-stranded DNA-enveloped virus that establishes latent and lifelong infections. Immunosuppression may trigger viral reactivation, leading to clinical illness [2]. It is an acute, highly contagious, and highly fatal disease characterized by sudden death, vascular damage and subsequent internal bleeding, lymphoid organ lesions, mucosal damage to the digestive tract, severe diarrhea, and parenchymal organ degeneration [3] The UL6 gene has the conserved region of the α-herpetic virus UL6 homologous gene, which provides the basis for the establishment of molecular biological diagnostic methods suitable for recessive infection DEV [4].

The goose parvovirus (GPV) outbreak in Jiangsu Province in 1956 caused a high mortality rate [5]. Ten years later, the virus has spread across Eurasia, causing serious damage to geese [6]. The virus could cause acute septicemic infectious disease in goslings, characterized by acute exudative enteritis, rapid transmission, rapid onset, and high mortality. The susceptible hosts of GPV range widely, including geese and Muscovy ducks [7,8]. From 2018 to 2021, 1970 samples were collected in 9 provinces in China, and the positive rate of GPV was as high as 12.74% [9]. Between 1982 and 1990, two parvovirus infections occurred in watery poultry in Taiwan, one caused by GPV and one by Muscovy duck parvovirus (MDPV). The nucleotide difference between GPV and MDPV was found to be from 16.2% to 19.4%. Due to the high similarity of nucleotides between the two viral pathogens, there may be high similarities in transmission routes and pathogenicity between GPV and MDPV [10]. The main characteristics of Muscovy duck parvovirus disease are diarrhea, motor dysfunction, developmental retardation, and death of young ducks. It is very similar to the symptoms of goose parvovirus disease [11]. Meanwhile, MDPV and GPV co-infection were detected in 7 of the 12 positive specimens in the previous study [12]. From the perspective of virology, physicochemical characteristics, and clinical symptoms, MDPV and GPV are difficult to distinguish; therefore, a rapid detection method is urgently needed to distinguish the two viruses.

With the ongoing expansion of waterfowl breed operations and increased stocking densities, DEV, GPV, and MDPV transmission has led to severe infections and substantial economic losses in the waterfowl industry [13]. Due to the similar host range of these three viruses, their ability to induce severe diarrhea in susceptible animals, and the frequent occurrence of co-infections, they pose significant challenges for clinical differentiation [14]. The similarity in clinical manifestations complicates diagnosis, necessitating the use of advanced molecular or serological techniques for precise differentiation and effective disease management. Probe-based quantitative polymerase chain reaction (qPCR) enables rapid quantification of multiple target gene expression levels [15]. In recent years, qPCR assays based on TaqMan probes have been successfully established for the simultaneous detection of MDPV and GPV [16]. In addition, a multiplex PCR method for the simultaneous detection of waterfowl parvovirus, duck enteritis virus, and goose astrocyte virus has been successfully established [17]. However, simultaneous detection of DEV, MDPV, and GPV based on TaqMan probes has not been reported. In this research, a multiplexed qPCR test for DEV, MDPV, and GPV was established, offering a technical approach for swift and effective detection and identification.

## 2. Materials and Methods

### 2.1. Primers and Probes

All available DEV (EF055890.1) (AY995224.1) (EF449516.1), MDPV (PQ820738.1) (PQ611596.1) (MH807445.1) (MH807698.1) (MH204100.1), and GPV (PV359174.1) (PQ536887.1) (MH717784.1) (MH209633.1) (PQ272760.1) sequences were obtained from GenBank and analyzed. According to the UL6 gene of DEV, the VP1 gene of MDPV, and the REP gene of GPV, the primers, and probes were designed using Oligo 6 and Primer Prime5 (5.0) software. Then, the obtained sequences were compared through NCBI BLAST to verify the specificity. Primers and probes were synthesized by Sangon Biotechnology Co., Ltd. (Shanghai, China) (Table 1).

### 2.2. Virus Strains and Clinic Samples

Virus strains used in this test, namely goose parvovirus (CVCC AV239), duck enteritis virus (CVCC AV18), muscovy duck parvovirus (MDPV), duck hepatitis A virus type 1 (DHAV-1), duck hepatitis A virus type 3 (DHAV-3), duck Tambusu virus (DTMUV), novel duck novel duck reovirus (NDRV), and avian influenza vaccine strain (FJ strain), Newcastle disease vaccine strain (La sota strain), are kept in our laboratory. We systematically collected clinical samples, including pharyngeal swabs, anal swabs, and organ samples (liver, lung, and heart) from waterfowl farms in Shandong, Jiangxi, and Fujian Provinces et al. in China.

### 2.3. Construction, Culture, and Purification of Standard Plasmids

The target fragments of DEV, MDPV, and GPV were amplified by PCR, then the PCR products were collected and purified, and the PCR fragments were cloned into the pMD-19T (Takara Biomedical Technology, Beijing, China) vector and confirmed by DNA sequencing (Sangon, Shanghai, China). Three recombinant plasmids, pMD19-DEV, pMD19-MDPV, and pMD19-GPV, were constructed. Following the guidelines of the TlANprep Mini Plasmid Kit (Tiangen Biotech, Beijing, China), the recombinant plasmids were extracted, and their concentrations were measured using a NanoDrop ND 2000 spectrophotometer (Thermo Scientific, Dreieich, Germany) and calculate the copy number.

(1) $$Plasmid\;length=\frac{\left(6.02\times 10^{23})\times (Xng/uL\times 10^{-9}\right)}{Plasmid\;length(bp)\times 660}$$

### 2.4. Optimization of Optimal Reaction Conditions for Multiplex qPCR

Amplification was performed using the Probe qPCR mix with UNG (Uracil N-Glycosylase) kit (Takara Biomedical Technology, Beijing, China). The total reaction system was 20 μL, and the experiment used 12.5 μL of Probe qPCR mix with UNG; primers, and probes at a molarity of 2–10 μM; and 2 μL of DNA mixture of the three pathogens. All reactions were performed on QuantStudio 7Flex (Thermo Fisher Scientific, Waltham, MA, USA). The reaction conditions were as follows: predenaturation at 94 °C for 3 min; denaturation at 94 °C for 5 s; annealing at 48–55 °C for 30 s; and extension at 72 °C 20 s for 40 cycles.

### 2.5. Construction of Standard Curves

To create a standard curve for DEV, MDPV, and GPV, each plasmid was serially diluted by a factor of ten, ranging from 10^7^ to 10^1^ copies/μL per microliter.

### 2.6. Sensitivity of Singleplex and Multiplex qPCR

The reaction conditions of single-plex qPCR and multiplex qPCR are the same. Tenfold serial dilutions of the standard plasmid were prepared in the range of 10^8^ to 10^0^ copies/μL. This range provides a comprehensive view of the analytical performance and ensures that it can accurately detect the presence of both high and low concentrations of viral DNA.

### 2.7. Specificity, and Repeatability of Multiplex qPCR

To evaluate the specificity of multiplex qPCR, the DNA/cDNA of DEV, GPV, MDPV, DTMUV, DHAV-3, NDRV, DHAV-1, AIV, and NDV as amplification templates. Nuclease-free water was used as the negative control template. To assess reproducibility, diluted templates were mixed in equal amounts experiments were performed and standard deviations within and between batches were calculated [18].

### 2.8. Sample Tested

A total 215 clinical samples were collected from geese and ducks with diarrhea and enteritis from different parts of China. All samples were added with 800 μL tirzol and grinding beads, grinding at 4 °C. Used TaKaRa MiniBEST Viral RNA and DNA Extraction Kit (Takara Biomedical Technology, Beijing, China) to extract nucleic acids from supernatants. All clinical samples were examined using the multiplex qPCR developed in this study.

## 3. Results

### 3.1. Optimization of Multiplex qPCR Reaction System

**Figure 1:**
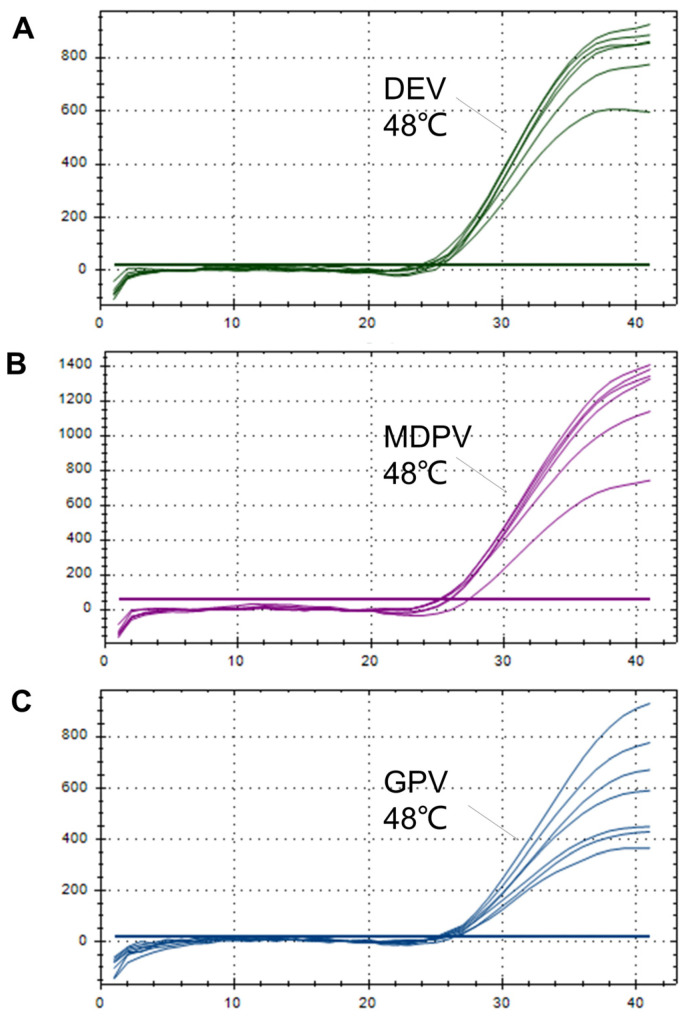
Optimization of annealing temperature for multiplex qPCR. During the annealing temperature optimization process, the annealing temperatures were set at 48, 50.2, 51.7, 53.1, 54, 54.6, and 55 °C, respectively. (**A**) Duck enteritis virus (DEV); (**B**), Muscovy duck parvovirus (MDPV); (**C**) Goose parvovirus (GPV).

The primer and probe molarity, along with the annealing temperature, were optimized through orthogonal experiments. The optimal annealing temperature was determined to be 48 °C (Figure 1). The reaction conditions were as follows: predenaturation at 94 °C for 3 min; denaturation at 94 °C for 5 s; annealing at 48 °C for 30 s; and extension at 72 °C 20 s for 40 cycles. Through the results of our experiments, we chose results with larger values of the longitudinal coordinates of the amplification curves, as well as lower Ct values at certain concentrations. As such, for DEV and MDPV, the molarity of the forward and reverse primers and the probe were 6 μM, 6 μM, and 8 μM, respectively. For GPV, the molarity of the forward and reverse primers and the probe were 8 μM, 8 μM, and 10 μM, respectively (Table 2 and Table 3).

### 3.2. Singleplex qPCR Detection of a Single Virus

**Figure 2:**
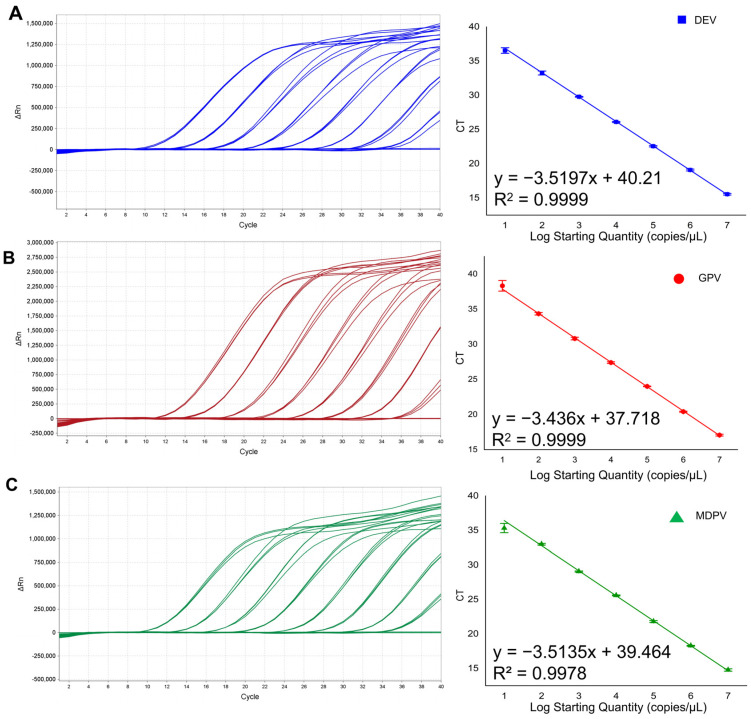
Establishment of singleplex qPCR assay for the individual virus. (**A**) The amplification curves (**left**) and a standard curve (**right**) for detection of DEV UL6 gene were generated concentrations ranging from 1.16 × 10^8^ to 1.16 × 10^0^ copies/μL, y = −3.5197x + 40.21; the coefficient of variation R^2^ = 0.99; E (amplification efficiency) = 92%; (**B**) The amplification curves (**left**) and a standard curve (**right**) for detection of GPV REP gene were generated concentrations ranging from 9.5 × 10^8^ to 9.5 × 10^0^ copies/μL, y = −3.436x + 37.718; the coefficient of variation R^2^ = 0.99; E = 95%; (**C**) The amplification curves (**left**) and a standard curve (**right**) for detection of MDPV VP1 gene was generated concentrations ranging from 1.48 × 10^8^ to 1.48 × 10^0^ copies/μL, y = −3.5135x + 39.464; the coefficient of variation R^2^ = 0.9978; E = 92%.

The established singleplex qPCR assays exhibited successful performance across all viruses: DEV demonstrated a detection limit of 11.6 copies, GPV reliably detected as low as 95 copies, and MDPV showcased a detection limit of 14.8 copies. The R-squared (R^2^) of DEV, MDPV, and GPV is above 0.99, and the amplification efficiency (Eff%) is above 90% (Figure 2). The results show that the individual qPCR assays for each virus were both effective and sensitive.

### 3.3. Establishment of a Multiplex qPCR Detection Assay

**Figure 3:**
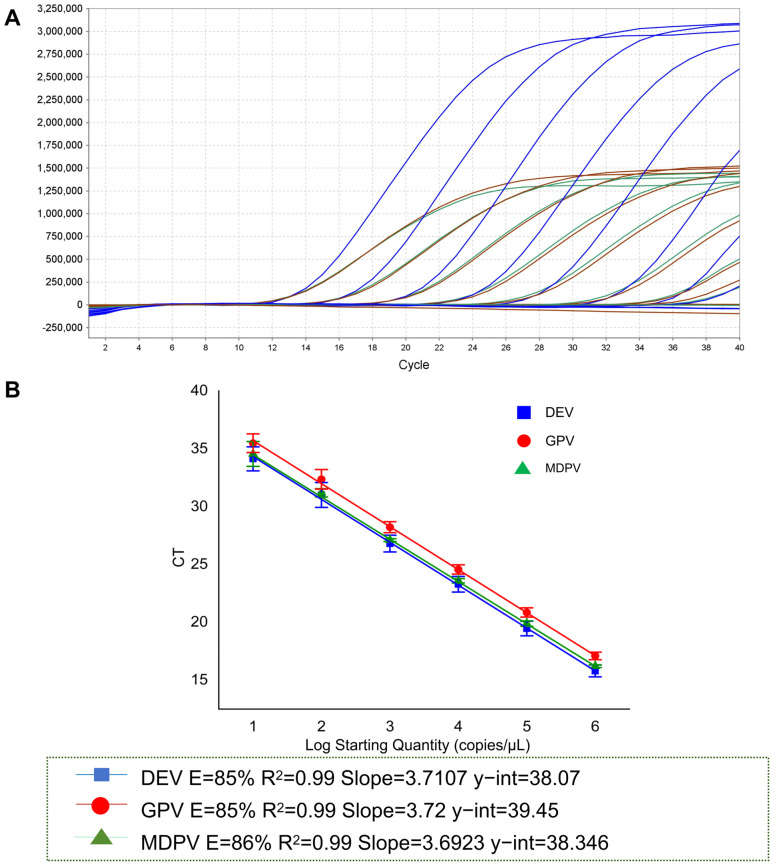
Establishment of multiplex qPCR sensitivity and standard curve. (**A**) The DEV, MDPV, and GPV amplification curves were generated using multiplex qPCR. Blue indicates the standard curve for DEV; green for MDPV; and red for GPV; (**B**) Standard curves of multiplex qPCR for detection of DEV, GPV, and MDPV were generated at the optimum amplification conditions. The standard curves of DEV (y = −3.7107x + 38.07 R^2^ = 0.99 E = 85%), GPV (y = −3.72x + 39.45 R^2^ = 0.99 E = 85%) and MDPV (y = −3.6923x + 38.346 R^2^ = 0.99 E = 86%).

The primer-probe molar concentrations used for the establishment of the multiplex qPCR method were consistent with those used in the single qPCR method. All the standard curves showed R^2^ and Eff%, with a good linear relationship between the Ct and the logarithms of the plasmid copy number. The R^2^ values of DEV, MDPV, and GPV were all above 0.99, and Eff% was 85 to 86 (Figure 3).

### 3.4. Sensitivity of Multiplex qPCR Detection

The results show that 116 copies of DEV, 14.8 copies of MDPV, and 95 copies of GPV were detected. Compared with a singleplex qPCR, the number of copies detected by DEV increased by 10^1^, as shown in Figure 3A and Table 4.

### 3.5. Specificity of Multiplex qPCR Detection

**Figure 4:**
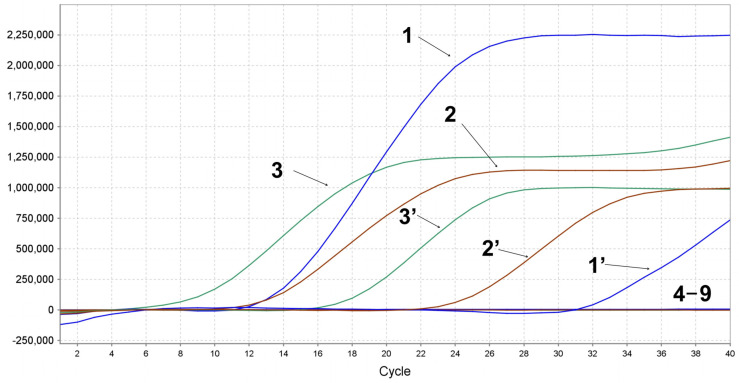
The specificity of multiplex qPCR. 1–3: plasmid templates of DEV, GPV, and MDPV, 1′–3′: nucleic acid templates of DEV, GPV, and MDPV, 4–9: nucleic acid templates of duck tambusu virus (DTMUV), novel duck reovirus (NDRV), duck hepatitis A type 1 virus (DHAV-1), duck hepatitis A type 3 virus (DHAV-3), avian influenza virus H9 (AIV H9), and Newcastle disease virus (NDV), and nuclease-free water control.

To assess the specificity of the multiplex qPCR assay, we used DNA and cDNA templates of DHAV-1, DHAV-3, NDRV, DTMUV, AIV, and NDV as amplification templates. The DEV, GPV, and MDPV DNA were successfully detected. No positive signals were detected for NDRV, DHAV-1, DHAV-3, DTMUV, AIV, and NDV, and the negative control, indicating the high specificity of the multiplex qPCR system based on TaqMan probes, as shown in Figure 4.

### 3.6. Repeatability of Multiplex qPCR Detection

As shown in Table 5, the DEV intra-assay test of CV was 0.51% to 2.28% and 0.28% to 1.12% for the inter-assay test. GPV amplification exhibited coefficients of variation of 0.34% to 2.17% and 0.24% to 1.12% for the intra-assay and inter-assay tests, respectively. MDPV amplification exhibited coefficients of variation of 0.42 to 2.26% and 0.55% to 1.10% for the intra- and inter-assay tests, respectively. The findings suggest that the TaqMan-probe-based multiplex qPCR assay developed in this research is repeatable and dependable.

### 3.7. Sample Tested

A total of 215 clinic samples exhibiting diarrhea and enteritis were collected from goose and duck farms in Anhui, Fujian, Guangdong, Guangxi, Shandong, Jiangxi, and Sichuan provinces across China. These samples were subsequently subjected to analysis using our multiplex qPCR techniques. As shown in Table 6 and Table 7, in 215 samples, the detection rate of DEV was the highest, and the rate of GPV and MDPV was 11.2–11.62%. Among them, the number of co-infections is small; the highest co-infection of DEV and GPV is 3.7%, whereas the detection rate of GPV and MDPV, DEV and MDPV is 1.4 to 2.8%. The detection rate of co-infection among the three viruses was 1.9%. Additionally, we performed a regional epidemiological analysis of the positive samples detected. DEV, MDPV, and GPV-positive samples were detected from southern China, which may be due to the largest proportion of duck farming in southern China.

## 4. Discussion

The widespread prevalence of DEV, MDPV, and GPV has significantly impacted waterfowl farming. The clinical diagnoses of these infectious diseases mainly depend on the observed characteristic clinical symptoms, such as lymphoid organ lesions caused by DEV infection, gastrointestinal mucosal injury, severe diarrhea, parenchymal organ degeneration; GPV and MDPV infection cause intestinal embolism, diarrhea, and pancreatic necrosis, diarrhea, and motor dysfunction, respectively [19]. The overlapping clinical manifestations of DEV, GPV, and MDPV infections necessitate the development of reliable molecular diagnostic assays to enable accurate pathogen differentiation and early clinical detection [13,20]. Current diagnostic approaches for DEV, MDPV, and GPV encompass conventional virus isolation, molecular techniques (LAMP, conventional PCR), and serological detection (ELISA) [21,22,23,24]. Existing DEV, MDPV, and GPV assays have limitations in terms of time, sensitivity, and specificity, so there is an urgent need for multiplex testing to improve diagnostic accuracy. TaqMan probe-based qPCR methods have high specificity and sensitivity, and with optimized probe channels, target genes can be detected multiplexed in a single-tube reaction [25]. For targeted detection, three pairs of specific primers and probes were designed, focusing on the UL6, VP1, and REP gene segments of DEV, MDPV, and GPV, respectively. However, multiple factors may affect amplification efficiency, such as deviations in annealing temperature that can lead to non-specific amplification [26]. Therefore, we optimize three key parameters, including annealing temperature, as well as primer and probe concentration. Firstly, by determining the annealing temperature as 48 °C, followed by orthogonal experiments on primer concentration and probe concentration, in the final results presented, the results with greater values of the longitudinal coordinates of the amplification curves, as well as lower values of Ct, were selected. At present, the detection assays for DEV, MDPV, and GPV mainly focus on the establishment of single qPCR assays and multiplex ordinary PCR. Studies show that the detection limit of DEV single ordinary PCR is 10^3^ copies, and the detection limit of MDPV and GPV simultaneously is 10^3^ copies [27,28]. In our study, the detection limit of DEV, MDPV, and GPV single qPCR is 10^1^ copies for the three targets, whereas the detection limit of multiplex qPCR is 10^2^, 10^1^, and 10^1^ copies, respectively. Among the common duck infectious diseases, aside from duck tambusu virus (DTMUV), novel duck reovirus (NDRV), duck hepatitis A type 1 virus (DHAV-1), duck hepatitis A type 3 virus (DHAV-3), avian influenza virus H9 (AIV H9), and Newcastle disease virus (NDV) have caused significant economic losses to the duck industry [29]. To verify the specificity of the method. We simultaneously detected nucleic acid templates of DEV, MDPV, GPV, DHAV-1, DHAV-3, DTMUV, NDRV, AIV, and NDV. The results show that DEV, MDPV, and GPV could be specifically detected and did not cross-react with other viruses. In the establishment of the multiplex qPCR method, repeatability detection is an important step to ensure the accuracy and reliability of experimental results. It has been shown that as the copy number gets smaller, the CV will be larger, and this value is valid when the CV is less than 10% [30]. Our results show that repeated detection of 10^3^,10^5^, and 10^7^ copies under the multiplex qPCR method had a CV of less than 3%. The results show that our multiplex qPCR assay has qualified reproducibility. Hence, the multiplex qPCR assay established in the current study demonstrated high sensitivity, robust specificity, and superior repeatability, offering substantial support for the clinical detection of DEV, MDPV, and GPV.

China is a major breeding country for waterfowl [31], and the main production areas are distributed in South China and Northeast China. Intensive breed practices not only facilitate virus transmission but also significantly contribute to viral genetic variation [32]. In our study, we collected 215 tissue samples from clinically suspected cases from seven provinces in China to explore the epidemiology of DEV, MDPV, and GPV. Epidemiological studies of DEV, MDPV, and GPV show that DEV was discovered in Yunnan, China, in 1998 and continues to be prevalent in many parts of China, especially in southern China [33]. GPV is distributed in both northern and southern China but varies according to the epidemic season in different regions [34]. In addition, MDPV is mainly prevalent in southern China, especially in Fujian Province [35]. In our study results, positive samples of DEV, MDPV, and GPV were mainly located in southern regions, with samples from the Fujian Province exhibiting detections of all three viruses, which is consistent with its endemic status. Meanwhile, we did not detect positive results for DEV and MDPV in the 30 samples from Shandong Province, which may be related to the endemic areas of these two viruses. In addition to the individual infections, we observed co-infections involving DEV and MDPV, GPV and MDPV, and GPV and DEV. Notably, there were also instances of co-infections involving DEV, GPV, and MDPV. Co-infections of GPV and MDPV have been documented in previous studies [18]. However, there have been no clinical reports of DEV and MDPV co-infections and co-infection of the three viruses. These findings reveal the intricate epidemiological dynamics and emphasized the critical need for comprehensive diagnostic strategies to manage effectively. In summary, we developed a multiplex qPCR for the simultaneous detection of DEV, GPV, and MDPV, providing an effective tool for epidemiological investigation.

## 5. Conclusions

The results show that the detection of DEV, MDPV, and GPV by specific primers and probes demonstrated high efficacy. In the 215 clinical samples, we detected various combinations including dual and triple co-infections of DEV and MDPV, DEV and GPV, MDPV and GPV, and DEV, MDPV and GPV. Our results further reveal the presence of mixed infections in waterfowl and highlighted the extent of mixed infections suggested. The multiplex qPCR method developed in this study provides a reliable, highly sensitive, and highly specific tool for early detection and simultaneous detection.

## Figures and Tables

**Table 1:** Primers and probes designed.

Virus	Primer and Probe	Sequence (5′-3′)	Target Gene	Size (bp)
DEV	Forward	GGCCAGGGAGTTTATAATTCGG	*UL6*	161
	Reverse	GCTATATGTCGTGCATCTAACCC		
	Probe	HEX-CTGCCATACGACAAATCCAGGCGAC-BHQ1		
MDPV	Forward	AAGCTACAACAACCACATCTAC	*VP1*	119
	Reverse	GGCAGTGGAATCTGTTGAAAT		
	Probe	CY5-ATCACAAGCGGAACAAACCCAGAC-BHQ2		
GPV	Forward	AATTGTTCTCATCAGTCGCTC	*REP*	153
	Reverse	AGTTTGCTTTCTCACATTCCATAC		
	Probe	FAM-CCTGTGACTCCTCAGAACTCCCCT-BHQ1		

**Table 2:** The Ct values of the primer-probe molarity of the optimized DEV, MDPV, and GPV.

Virus		Primer (μM)	2	4	6	8	10
	Probe (μM)						
DEV	2		24.49	24.30	25.28	24.80	24.78
	4		25.38	24.44	24.56	27.54	24.85
	6		23.96	24.50	23.86	24.66	24.64
	8		24.10	25.01	23.44	24.37	23.59
	10		24.41	24.58	24.12	24.83	24.57
MDPV	2		24.99	25.28	25.68	25.88	25.71
	4		24.30	24.08	25.17	25.15	25.42
	6		24.81	25.13	25.20	25.20	25.15
	8		24.96	25.01	24.12	24.37	23.59
	10		24.41	24.58	24.26	24.83	24.36
GPV	2		26.71	26.34	26.92	27.52	27.44
	4		24.86	24.82	26.97	26.24	27.50
	6		25.77	25.79	26.55	27.04	27.60
	8		25.23	25.80	25.82	25.84	25.38
	10		24.64	22.73	25.33	26.13	24.93

**Table 3:** Molarity of primers and probes in multiplex qPCR reaction system.

Primer and Probe	Molarity (μM)		
	DEV	MDPV	GPV
Forward (μM)	6	6	8
Reverse (μM)	6	6	8
Probe (μM)	8	8	10

**Table 4:** Sensitivity of multiplex qPCR.

Templates (Copies/Gene)	Ct Value								
	DEV			MDPV			GPV		
10^8^	12.65	12.63	11.88	12.88	12.69	12.88	13.95	13.75	13.33
10^7^	16.10	16.08	15.20	16.34	16.12	16.15	17.39	17.13	16.75
10^6^	19.83	19.81	18.73	20.14	19.85	19.68	21.21	20.89	20.42
10^5^	23.52	23.81	22.52	23.65	23.74	23.37	24.81	24.78	24.10
10^4^	27.12	27.31	25.97	27.19	27.16	26.97	28.50	28.48	27.67
10^3^	31.41	31.82	29.78	31.37	31.44	30.79	32.86	32.84	31.38
10^2^	33.14	35.22	34.03	33.32	35.13	35.23	34.84	36.38	35.23
10^1^	ND			38.86	38.90	38.83	37.41	38.91	38.77
NTC	ND			ND			ND		

ND: Not Detected.

**Table 5:** Repeatability test of the multiplex qPCR.

Plasmid Standards	Concentration (Copies/μL)	Intra-Assay Ct Values			Inter-Assay Ct Values		
		$\bar{x}$	SD	CV (%)	$\bar{x}$	SD	CV (%)
pMD19-DEV	10^7^	16.08	0.087	0.54	15.79	0.085	0.54
	10^5^	23.81	0.543	2.28	23.28	0.26	1.12
	10^3^	31.82	0.161	0.51	31.01	0.087	0.28
pMD19-MDPV	10^7^	16.11	0.09	0.56	16.20	0.133	0.82
	10^5^	23.70	0.535	2.26	23.58	0.260	1.10
	10^3^	31.43	0.132	0.42	31.19	0.172	0.55
pMD19-GPV	10^7^	17.131	0.136	0.79	17.091	0.142	0.83
	10^5^	24.784	0.538	2.17	24.567	0.274	1.12
	10^3^	32.849	0.111	0.34	32.364	0.078	0.24

$\bar{x}$: Take the average of the Ct values; SD (standard deviation): √(Σ(*xi* − μ)^2^/N); CV (coefficient of variation): (Standard deviation/Mean) × 100%.

**Table 6:** Positive rate of DEV, GPV, and MDPV in clinical samples by qPCR.

Virus	Total Clinical Samples	Positive Rate (%)
		qPCR
DEV	215	33/215 (15.3%)
GPV	215	25/215 (11.6%)
MDPV	215	24/215 (11.2%)
DEV and MDPV	215	6/215 (2.8%)
GPV and MDPV	215	3/215 (1.4%)
GPV and DEV	215	8/215 (3.7%)
DEV, MDPV and GPV	215	4/215 (1.9%)

**Table 7:** Multiplex qPCR assays were employed to analyze clinical samples obtained from various geographical regions.

Province	Number	Number of Positive Samples		
		DEV	GPV	MDPV
Anhui	12	0	3	3
Fujian	71	7	11	15
Guangdong	23	0	2	0
Guangxi	33	15	1	6
Shandong	30	0	6	0
Jiangxi	43	10	1	0
Sichuan	3	1	1	0

## Data Availability

The original contributions presented in this study are included in the article. Further inquiries can be directed to the corresponding author.

## References

[1] Wang G., Qu Y., Wang F., Hu D., Liu L., Li N., Yue R., Li C., Liu S. (2013). The comprehensive diagnosis and prevention of duck plague in northwest Shandong province of China. Poult. Sci..

[2] Cohen J.I. (2024). Therapeutic vaccines for herpesviruses. J. Clin. Investig..

[3] Xie L., Xie Z., Huang L., Wang S., Huang J., Zhang Y., Zeng T., Luo S. (2017). A polymerase chain reaction assay for detection of virulent and attenuated strains of duck plague virus. J. Virol. Methods.

[4] Li H., Liu S., Han Z., Shao Y., Chen S., Kong X. (2009). Comparative analysis of the genes UL1 through UL7 of the duck enteritis virus and other herpesviruses of the subfamily alphaherpesvirinae. Genet. Mol. Biol..

[5] Fan W., Sun Z., Shen T., Xu D., Huang K., Zhou J., Song S., Yan L. (2017). Analysis of evolutionary processes of species jump in waterfowl parvovirus. Front. Microbiol..

[6] Gough R.E., Spackman D. (1982). Studies with a duck embryo adapted goose parvovirus vaccine. Avian Pathol..

[7] Wozniakowski G., Samorek-Salamonowicz E., Kozdrun W. (2012). Quantitative analysis of waterfowl parvoviruses in geese and muscovy ducks by real-time polymerase chain reaction: Correlation between age, clinical symptoms and DNA copy number of waterfowl parvoviruses. BMC Vet. Res..

[8] Yu J., Zou J., Liu X., Pan Y., Mu Y., Li S., Wang J., Xu F., Wang Y. (2023). TaqMan-probe-based multiplex real-time RT-qPCR for simultaneous detection of GoAstV, GPV, and GoCV. Poult. Sci..

[9] He D., Wang F., Zhao L., Jiang X., Zhang S., Wei F., Wu B., Wang Y., Diao Y., Tang Y. (2022). Epidemiological investigation of infectious diseases in geese on mainland China during 2018–2021. Transbound. Emerg. Dis..

[10] Chang P.C., Shien J.H., Wang M.S., Shieh H.K. (2000). Phylogenetic analysis of parvoviruses isolated in taiwan from ducks and geese. Avian Pathol..

[11] Wang J., Huang Y., Zhou M., Hardwidge P.R., Zhu G. (2016). Construction and sequencing of an infectious clone of the goose embryo-adapted muscovy duck parvovirus vaccine strain FZ91-30. Virol. J..

[12] Dong J., Bingga G., Sun M., Li L., Liu Z., Zhang C., Guo P., Huang Y., Zhang J. (2019). Application of high-resolution melting curve analysis for identification of muscovy duck parvovirus and goose parvovirus. J. Virol. Methods.

[13] Yang Y., Sui N., Zhang R., Lan J., Li P., Lian C., Li H., Xie Z., Jiang S. (2020). Coinfection of novel goose parvovirus-associated virus and duck circovirus in feather sacs of cherry valley ducks with feather shedding syndrome. Poult. Sci..

[14] Zhang H., Han F., Shu X., Li Q., Ding Q., Hao C., Yan X., Xu M., Hu H. (2022). Co-infection of porcine epidemic diarrhoea virus and porcine deltacoronavirus enhances the disease severity in piglets. Transbound. Emerg. Dis..

[15] Hawkins S., Guest P.C. (2017). Multiplex analyses using real-time quantitative PCR. Methods Mol. Biol..

[16] Wan C., Chen C., Cheng L., Liu R., Shi S., Fu G., Chen H., Fu Q., Huang Y. (2019). Specific detection and differentiation of classic goose parvovirus and novel goose parvovirus by TaqMan real-time PCR assay, coupled with host specificity. BMC Vet. Res..

[17] Dai Y., Li M., Hu X., Zhao R., Xia L. (2022). Development and application of a multiplex PCR method for simultaneous detection of waterfowl parvovirus, duck enteritis virus and goose astrovirus. 3 Biotech.

[18] Wang H., Chen J., An T., Chen H., Wang Y., Zhu L., Yu C., Xia C., Zhang H. (2024). Development and application of quadruplex real time quantitative PCR method for differentiation of muscovy duck parvovirus, goose parvovirus, duck circovirus, and duck adenovirus 3. Front. Cell. Infect. Microbiol..

[19] Liang Z., Guo J., Yuan S., Cheng Q., Zhang X., Liu Z., Wang C., Li Z., Hou B., Huang S. (2022). Pathological and molecular characterization of a duck plague outbreak in southern China in 2021. Animals.

[20] Liu M., Zhao Y., Hu D., Huang X., Xiong H., Qi K., Liu H. (2019). Clinical and histologic characterization of co-infection with astrovirus and goose parvovirus in goslings. Avian Dis..

[21] Qi X., Yang X., Cheng A., Wang M., Guo Y., Jia R. (2009). Replication kinetics of duck virus enteritis vaccine virus in ducklings immunized by the mucosal or systemic route using real-time quantitative PCR. Res. Vet. Sci..

[22] Yang X., Qi X., Cheng A., Wang M., Zhu D., Jia R., Chen X. (2010). Intestinal mucosal immune response in ducklings following oral immunisation with an attenuated duck enteritis virus vaccine. Vet. J..

[23] Wang K., Wang C.J., Pan L., Wang G.J., Qi K.Z., Liu H.M. (2016). Isolation and characterization of a goose parvovirus from yan goose. Acta Virol..

[24] Wan C., Cheng L., Chen C., Liu R., Shi S., Fu G., Chen H., Fu Q., Huang Y. (2019). A duplex PCR assay for the simultaneous detection and differentiation of muscovy duck parvovirus and goose parvovirus. Mol. Cell. Probes.

[25] Gadkar V., Filion M. (2014). New developments in quantitative real-time polymerase chain reaction technology. Curr. Issues Mol. Biol..

[26] Rychlik W., Spencer W.J., Rhoads R.E. (1990). Optimization of the annealing temperature for DNA amplification in vitro. Nucleic Acids Res..

[27] Wan C., Shi S., Chen C., Chen H., Cheng L., Fu Q., Fu G., Liu R., Huang Y. (2018). Development of a PCR assay for detection and differentiation of muscovy duck and goose parvoviruses based on NS gene characterization. J. Vet. Med. Sci..

[28] Yao M., Zhang X., Gao Y., Song S., Xu D., Yan L. (2019). Development and application of multiplex PCR method for simultaneous detection of seven viruses in ducks. BMC Vet. Res..

[29] Zhang Y.F., Xie Z.X., Xie L.J., Deng X.W., Xie Z.Q., Luo S.S., Huang L., Huang J.L., Zeng T.T. (2015). GeXP analyzer-based multiplex reverse-transcription PCR assay for the simultaneous detection and differentiation of eleven duck viruses. BMC Microbiol..

[30] Taylor S.C., Nadeau K., Abbasi M., Lachance C., Nguyen M., Fenrich J. (2019). The ultimate qPCR experiment: Producing publication quality, reproducible data the first time. Trends Biotechnol..

[31] Lu L., Chen Y., Wang Z., Li X., Chen W., Tao Z., Shen J., Tian Y., Wang D., Li G. (2015). The goose genome sequence leads to insights into the evolution of waterfowl and susceptibility to fatty liver. Genome Biol..

[32] Volz E.M., Koelle K., Bedford T. (2013). Viral phylodynamics. PLoS Comput. Biol..

[33] Kong J., Feng K., Zhao Q., Chen Y., Wang J., Chen S., Shao G., Liao L., Li Y., Xie Z. (2022). Pathogenicity and transmissibility studies on live attenuated duck enteritis virus vaccine in non-target species. Front. Microbiol..

[34] Huo X., Chen Y., Zhu J., Wang Y. (2023). Evolution, genetic recombination, and phylogeography of goose parvovirus. Comp. Immunol. Microbiol. Infect. Dis..

[35] Zhao H., Xie Z., Xie L., Deng X., Xie Z., Luo S., Huang L., Huang J., Zeng T. (2014). Molecular characterization of the full muscovy duck parvovirus, isolated in Guangxi, China. Genome Announc..

